# The function of a 60-nm-thick AlN buffer layer in n-ZnO/AlN/p-Si(111)

**DOI:** 10.1186/s11671-015-0809-3

**Published:** 2015-02-28

**Authors:** Wei Wang, Chao Chen, Guozhen Zhang, Ti Wang, Hao Wu, Yong Liu, Chang Liu

**Affiliations:** Key Laboratory of Artificial Micro- and Nano-structures of Ministry of Education, and School of Physics and Technology, Wuhan University, Wuhan, 430072 People’s Republic of China

**Keywords:** AlN buffer layer, Heterojunction, ALD, Band alignment

## Abstract

ZnO films were prepared on p-Si (111) substrates by using atomic layer deposition. High-resolution x-ray diffraction (XRD), scanning electron microscopy (SEM), x-ray photoelectron spectroscopy (XPS), photoluminescence (PL), and I-V measurements were carried out to characterize structural, electrical, and optical properties. After introducing a 60-nm-thick AlN buffer layer, the growth direction of the ZnO films was changed from [10] to [0002]. Meanwhile, the ZnO crystalline quality was significantly improved as verified by both XRD and PL analyses. It has been demonstrated that the reverse leakage current was greatly reduced with the AlN buffer layer. The valence band offsets have been determined to be 3.06, 2.95, and 0.83 eV for ZnO/Si, ZnO/AlN, and AlN/Si heterojunctions, respectively, and the band alignment of ZnO/Si heterojunction was modified to be 0.72 eV after introducing the AlN buffer layer. Our work offered a potential way to fabricate Si-based ultraviolet light-emitting diodes and improve the device performances.

## Background

Si is the most useful substrate due to its low cost and easy integration [1,2]. P-type Si has the prominent merits to make driving voltage of light-emitting diode (LED) lower and the cost of device less expensive [3]. Therefore, the Si-based LEDs possess great competitiveness. Zinc oxide (ZnO) is of interest as a result of its potential applications in ultraviolet (UV) optoelectronic devices due to its wide band gap of approximately 3.4 eV at room temperature and high exciton binding energy of approximately 60 meV [4]. Moreover, ZnO is thermally and chemically stable in ambient air, highly transparent in the visible region (>85%) [5], resistant to be oxidized, easy to fabricate, and relatively cheap compared to other optoelectronic materials [6]. So far, a variety of techniques have been utilized to grow ZnO thin films, such as, sputtering [7], molecular beam epitaxy (MBE) [8], pulsed laser deposition [9], and chemical vapor deposition [10]. As a chemical vapor processing with accurate surface control and self-limiting, atomic layer deposition (ALD) can prepare thin films with high uniformities and low defect densities.

Because of the large mismatches of the lattice constants (15.4%) and thermal expansion coefficients (60%) between ZnO and Si [11] and the oxidation of Si surface, direct growth of ZnO on Si substrates resulted in amorphous or polycrystalline films [12]. Attempts have been made to solve this problem by inserting different buffer layers between ZnO and substrates, such as GaN [13] and Al_2_O_3_ [14,15]. It is well know that in the system of n-ZnO/p-Si, electron and hole recombination takes place mainly in Si rather than in ZnO, leading to a difficult application for lighting since Si has a narrow and indirect band gap. Owing to the difference in band gaps between ZnO and Si that provides an effective electron injection from ZnO to p-Si and blocks the flow of holes from p-Si to ZnO [16], it is necessary to understand the physical properties of the ZnO/Si heterojunction, especially the energy band alignment and interfacial microstructure. Some researchers employed different dielectric buffer layers to study the band alignment of the ZnO/p-Si heterojunction [16-18]. However, few reports mentioned AlN buffer layers between ZnO and Si. AlN has the widest direct band gap (6.2 eV) among all III-nitride semiconductors [19] and is used as buffer layer to grow GaN due to its outstanding physical and chemical properties including high insulating resistance, high stability in severe conditions, and high thermal conductivity [20].

In this work, AlN buffer layer was introduced and the valence band offset (VBO) of ZnO/Si heterojunction was modified from 3.06 to 3.78 eV while the VBO between AlN and Si substrate was 2.95 eV. It was reported that the presence of SiO_x_ made the VBO between SiO_x_ and Si to be 3.15 eV [17], and those of Al_2_O_3_ and HfO_2_ between ZnO and Si substrate made the VBOs to be 3.24 and 2.87 eV, respectively [16].

Comparing with (100)-ZnO, (002)-ZnO exhibits better thermal stability. Therefore, control of the growth direction of ZnO is crucial. Pung et al. [21] have proved that the deposition temperature played an important role in preferential growth of ZnO films, but changing the growth direction of ZnO films by inserting different buffer layers was rarely studied. In the early work of our group, we have prepared n-type (100)-ZnO deposited on Si (111) substrates with and without an Al_2_O_3_ buffer layer by ALD [14]. Under the same growth conditions, the growth direction of ZnO films has changed from [100] to [002] after introducing AlN as the buffer. The crystallization of the ZnO films was clearly improved, and the leakage current was significantly reduced.

## Methods

The p-Si (111) substrates used in this work were all dipped for 60 s in a dilute aqueous hydrofluoric acid solution (2% HF) for the removal of the native oxide. The wafers were then ultrasonically cleaned in acetone for 10 min to remove organic grease and rinsed with ethanol.

The AlN buffer layer was grown by MBE (SVTA 35 V-2). The growth temperature and Al source temperature were set at 800°C and 1260°C, respectively. Prior to the growth, the Si substrate was thermally cleaned at 850°C for 10 min. Subsequent nitridation was performed at 850°C for 10 min in a nitrogen atmosphere with a flow rate of 2.65 sccm under 500 W RF-plasma power. The growth time for AlN was 5 min.

ZnO films were then deposited on AlN buffer layer by ALD (Beneq TFS-200) at a temperature of 200°C. Diethyl zinc (DEZn) and deionized water (H_2_O) were used as the sources of zinc and oxygen, respectively. During the deposition, DEZn and H_2_O were alternately fed into the chamber by using nitrogen as the carrier gas. A purge process using nitrogen was also introduced to clean the redundant former precursor. Four thousand cycles were performed and the ZnO films were 600 nm thick as measured by ellipsometer (Alpha-SE, J.A. Woollam, Lincoln, USA). A comparison between two samples named as A (without AlN buffer layer) and B (with an AlN buffer layer about 60 nm) was performed to assess the function of the buffer layer

High-resolution x-ray diffraction (HRXRD, Bede D1, Bede Scientific Instruments, Durham, England, UK) and scanning electron microscope (SEM, Hitachi S-4800, Hitachi, Tokyo, Japan) were used to study the crystallization and interfacial properties. Photoluminescence (PL, BITPE miniPL-5.5, Photon Systems, Covina, CA, USA) measurements were carried out to analyze the near-band-edge (NBE) and deep emissions of the ZnO films with or without the AlN buffer layer. The electrical measurements were carried out to compare the effects of the buffer layer. The energy band alignments of ZnO/Si heterojunction with or without the AlN buffer layer were measured by x-ray photoelectron spectroscopy (XPS; Thermo Scientific ESCLAB 250Xi, Thermo Fisher Scientific, Waltham, MA, USA).

For the XPS experiments, seven samples were prepared, namely, (1) a clean Si substrate; (2) a 5 nm ZnO film on Si substrate; (3) a 600-nm-thick ZnO film grown on Si substrate; (4) a 5-nm-thick AlN film grown on Si substrate; (5) a 60-nm-thick AlN film grown on Si substrate; (6) a ZnO (5 nm)/AlN (60 nm) heterostructure grown on Si substrate; (7) a ZnO (600 nm)/AlN (60 nm) heterostructure grown on Si substrate. The 5-nm-thick AlN layer was grown by MBE with the same conditions mentioned above but the growth period was 30 s.

## Results and discussion

**Figure 1:**
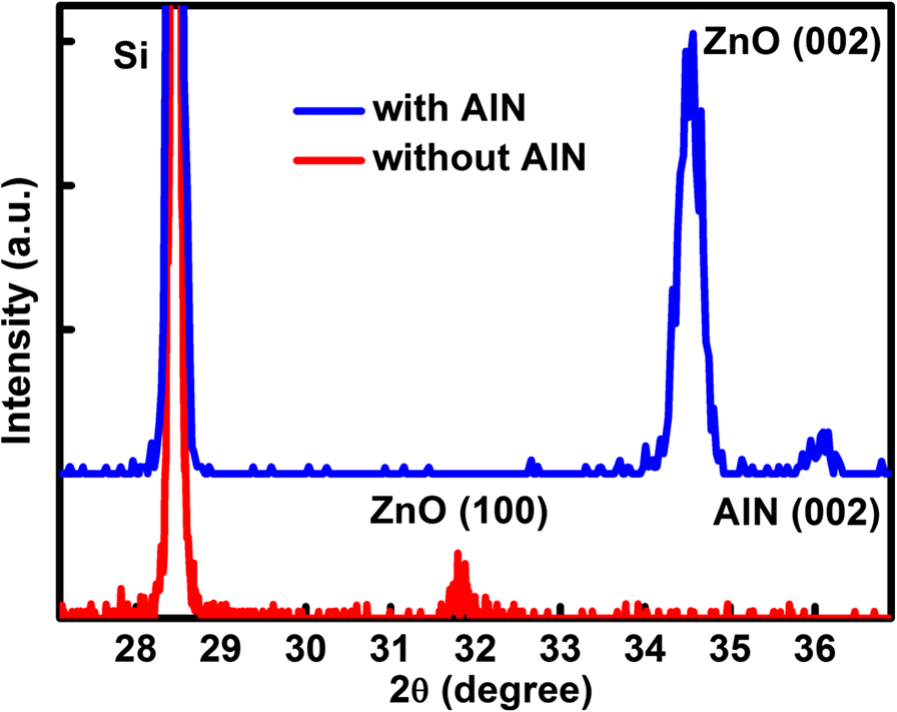
**The Omega-2theta scans of XRD spectra of sample a (without AlN) and sample b (with AlN).**

Figure 1 shows the XRD spectra of ZnO thin films grown by ALD without (sample A) and with the AlN buffer layer (sample B). For both samples, the diffraction peaks were all matched with the standard diffraction pattern of ZnO and AlN. As depicted in Figure 1, it was clearly seen that the thin AlN buffer layer played an important role in determining the crystalline orientation of the ZnO thin films.

For sample A, the growth direction of ZnO film was [100], indicating the nonpolar nature of the ZnO films [14]. During the growth at 200°C, DEZn may dissociate into ethyl group fragments such as CH_3_ and these anions might adhere to the positively charged Zn-(002) polar surface [21,22]. Therefore, the growth direction of ZnO films was [100] on Si. On the other side, the lattice mismatch between AlN and ZnO was only 3.8% (calculated from the powder diffraction file card 89–0510 and 89–3446 for ZnO and AlN, respectively), which was much less than that between ZnO and Si substrate. Thus, when the AlN buffer layer was introduced, the growth direction of ZnO films was kept along [002]. This is quite similar to what happened when InGaN buffer layer was introduced between ZnO and Si substrate [23].

Meanwhile, the intensity of ZnO (002) peak for sample B was much stronger than that of ZnO (100) peak for sample A. Considering that the ZnO films were deposited and tested under the identical conditions, it demonstrated that the AlN buffer layer forced ZnO films to grow along the c-axis.

**Figure 2:**
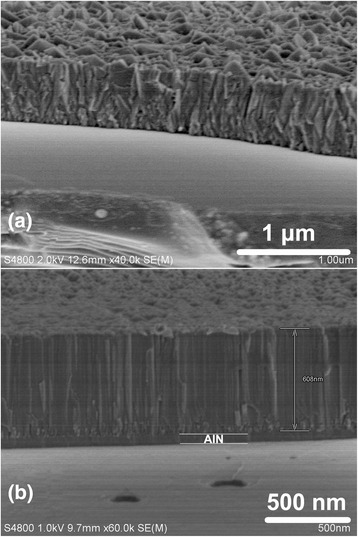
**Cross-sectional SEM images of sample a and b.**

The cross-sectional SEM images of samples A and B are shown in Figure 2. The irregular cross profile of sample A revealed a polycrystalline nature of the ZnO film grown without any buffer layer on Si. Figure 2b shows that the AlN buffer layer was introduced between the ZnO film and Si substrate, and the thickness of AlN buffer layer was measured to be 60 nm. It is obvious that the ZnO films in Figure 2b was regularly oriented while it was rambled in Figure 2a. The number of stacking faults and edge dislocations of sample B were obviously less than that of sample A. It can be concluded that with the AlN buffer layer, the crystalline quality of ZnO films was significantly improved.

**Figure 3:**
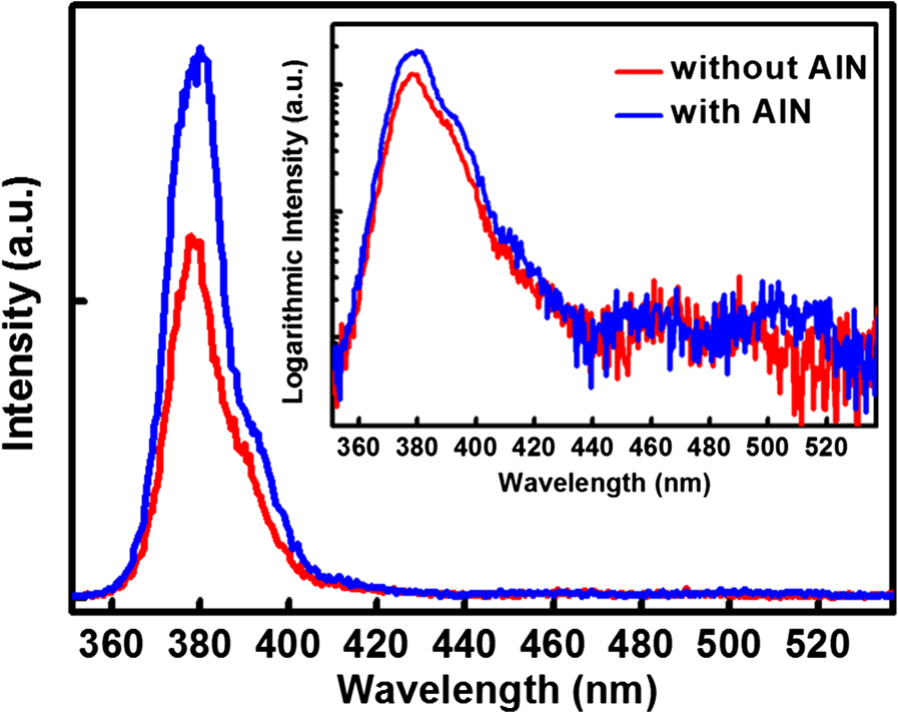
**The room temperature PL spectra of samples a (without AlN) and b (with AlN).** The inset shows the logarithmic intensity spectra.

The room temperature PL spectra of ZnO thin films with and without the AlN buffer layer are shown in Figure 3. The main PL peaks at about 380 nm were due to the free excitation emission in ZnO, and a low energy shoulder (at wavelength of 395 nm) was attributable to the emission related to point defects [24]. Usually, the ratio of band-edge transition (BET) to deep level emissions (DLEs) could evaluate the quality of ZnO films. It has been shown that the quality of ZnO films has been significantly improved after inserting the AlN buffer layer, because the ratio of BET to DLE of sample B (59.8) was larger than that of sample A (43.4). The DLE around 500 nm was caused by different intrinsic defects in ZnO films such as oxygen vacancies and zinc interstitials [14]. The very weak DLEs of samples A and B suggest that few zinc interstitials and few oxygen vacancies exist in the ZnO films, which can be attributed to the growth mechanism of ALD. The self-limiting aspect of ALD leads to a conformal deposition, because the precursors can adsorb and subsequently desorb from the surface where the reaction has reached completion, and then proceed to react with other unreacted surface areas, while the redundant former precursor should be cleaned by the purge process. In this way, the two reactions (ZnOH^*^ and Zn(CH_2_CH_3_) in this work) proceeded in a sequential fashion to deposit a thin film with atomic level control [25].

This can be further understood through the growth procedures of ALD as shown in the following reactions [21]:

$$ \begin{array}{c}\hfill ZnO{H}^{*}+Zn{\left(C{H}_2C{H}_3\right)}_2\to ZnOZn{\left(C{H}_2C{H}_3\right)}^{*}+{C}_2{H}_6\hfill \\ {}\hfill Zn{\left(C{H}_2C{H}_3\right)}^{*}+{H}_2O\to ZnO{H}^{*}+{C}_2{H}_6\hfill \end{array} $$

**Figure 4:**
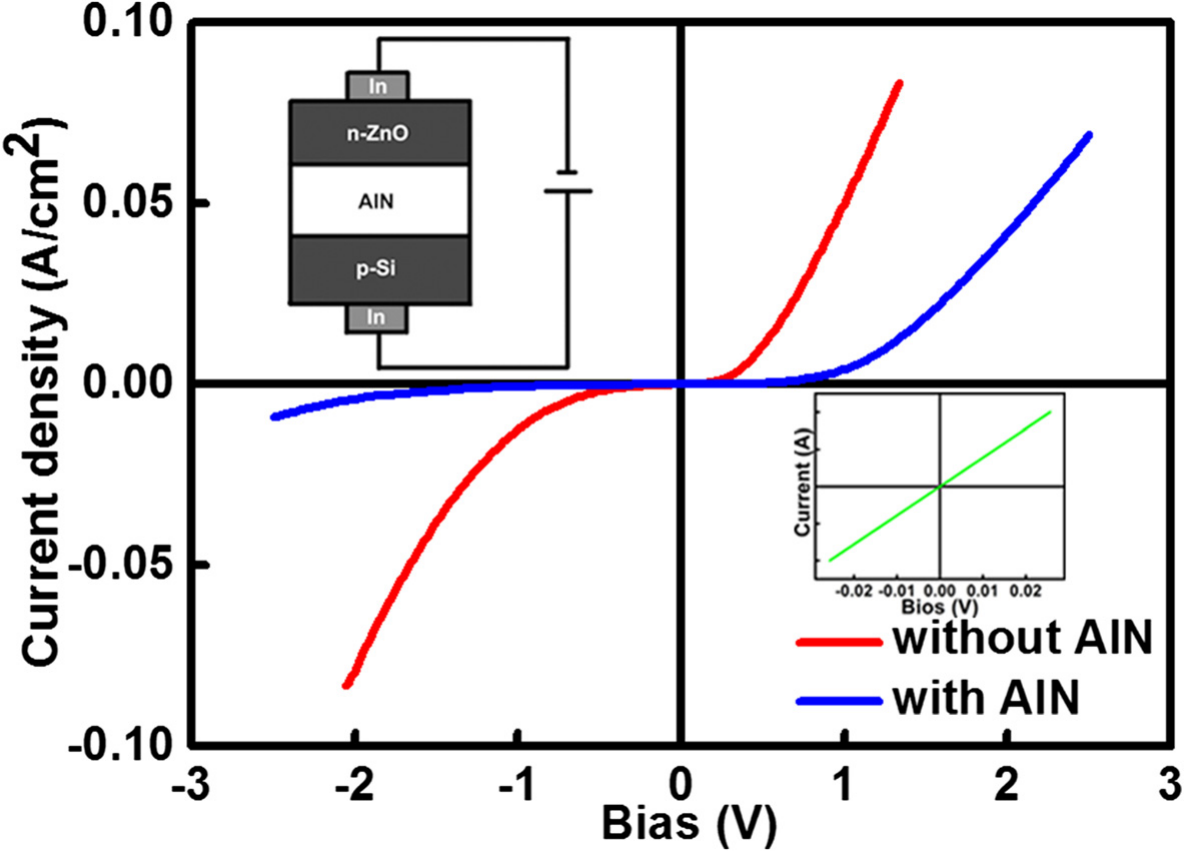
**I to V curves of the samples a (without AlN) and b (with AlN).** A rectification behavior can be observed. The inset shows the sketch map of our measurement configuration and ohmic contacting I to V curves of the indium electrodes on the surface of Si substrate.

Figure 4 shows a current–voltage (I to V) characteristic of the n-ZnO/AlN/p-Si heterostructure. The indium electrodes were welded on the surfaces of ZnO and Si layers to achieve the ohmic contacts. The ZnO films were etched to 1 mm × 1 mm by lithographic process to reduce contact resistance. The I to V curves were measured by changing the bias voltage from +3 to −3 V. The current was restricted between 100 and −100 mA to protect the devices. Diode-like rectification behaviors were observed. Under the reverse bias of −2 V, the leakage current of the n-ZnO/p-Si without the AlN buffer layer was about 94 mA (sample A) while that with the buffer (sample B) was about only 6 mA. Thus, the leakage current has been effectively reduced due to the excellent insulating property of the AlN buffer layer. Under the forward biases, the turn-on voltage of the sample A was about 0.47 V while it increased to about 1.25 V for sample B. The increase of the threshold voltage was also due to the introduction of the insulating AlN buffer layer.

The energy band alignment of ZnO/Si and ZnO/AlN/Si heterojunctions has been studied by using x-ray photoelectron spectroscopy (XPS). The VBO of ZnO/Si heterojunction can be calculated according to Kraut’s method [26] as follows:

1 $$ \varDelta {E}_{\mathrm{V}}^{\mathrm{Zn}\mathrm{O}/\mathrm{S}\mathrm{i}}=\left({E}_{{}_{\mathrm{Si}2\mathrm{s}}}^{\mathrm{Si}}-{E}_{\mathrm{V}\mathrm{BM}}^{\mathrm{Si}}\right)-\left({E}_{\mathrm{Zn}2{\mathrm{p}}_{3/2}}^{\mathrm{Zn}\mathrm{O}}-{E}_{VBM}^{ZnO}\right)-\varDelta {E}_{\mathrm{CL}} $$

2 $$ \varDelta {E}_{\mathrm{V}}^{\mathrm{Al}\mathrm{N}/\mathrm{Z}\mathrm{n}\mathrm{O}}=\left({E}_{\mathrm{Zn}2{\mathrm{p}}_{3/2}}^{\mathrm{Zn}\mathrm{O}}-{E}_{\mathrm{V}\mathrm{BM}}^{\mathrm{Zn}\mathrm{O}}\right)-\left({E}_{\mathrm{Al}2{\mathrm{p}}_{3/2}}^{\mathrm{Al}\mathrm{N}}-{E}_{\mathrm{V}\mathrm{BM}}^{\mathrm{Al}\mathrm{N}}\right)-\varDelta {E}_{\mathrm{CL}1} $$

3 $$ \varDelta {E}_{\mathrm{V}}^{\mathrm{Al}\mathrm{N}/\mathrm{S}\mathrm{i}}=\left({E}_{{}_{\mathrm{Si}2\mathrm{s}}}^{\mathrm{Si}}-{E}_{\mathrm{V}\mathrm{BM}}^{\mathrm{Si}}\right)-\left({E}_{\mathrm{Al}2{\mathrm{p}}_{3/2}}^{\mathrm{Al}\mathrm{N}}-{E}_{\mathrm{V}\mathrm{BM}}^{\mathrm{Al}\mathrm{N}}\right)-\varDelta {E}_{\mathrm{CL}2} $$

Where ${E}_{\mathrm{i}}^{\mathrm{s}}$ denotes the energy for feature i in sample s, Zn 2p_3/2_, Al 2p_3/2_, and Si 2 s core levels (CLs) were used in Equations 1 to 3. The CL spectra were fitted by the Voigt (mixed Gaussian-Lorentzian) line shape by employing a Shirly background to determine the respective CL positions. *ΔE*CL, *ΔE*CL_1_, and *ΔE*CL_2_ were the energy difference between the CLs of Zn 2p_3/2_ and Si 2 s, Zn 2p_3/2_ and Al 2p_3/2_, and Si 2 s and Al 2p_3/2_, respectively.

**Figure 5:**
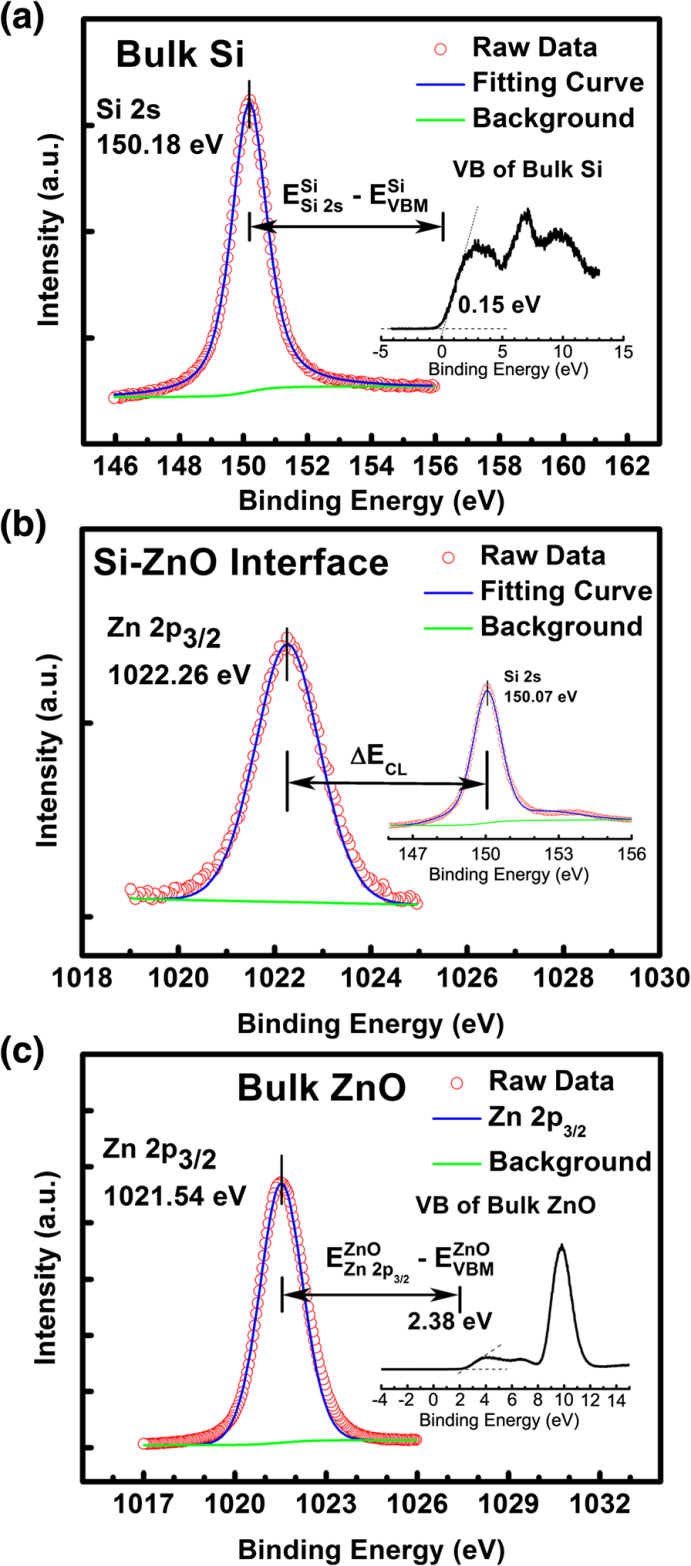
**XPS narrow scans of ZnO/Si heterojunction. (a)** XPS narrow scans of Si 2 s and VBM of bulk Si; **(b)** XPS narrow scans of Zn 2p3/2 and Si 2 s at ZnO/Si interface; **(c)** XPS narrow scans of Zn 2p3/2 and VBM of bulk ZnO on a Si substrate.

**Figure 6:**
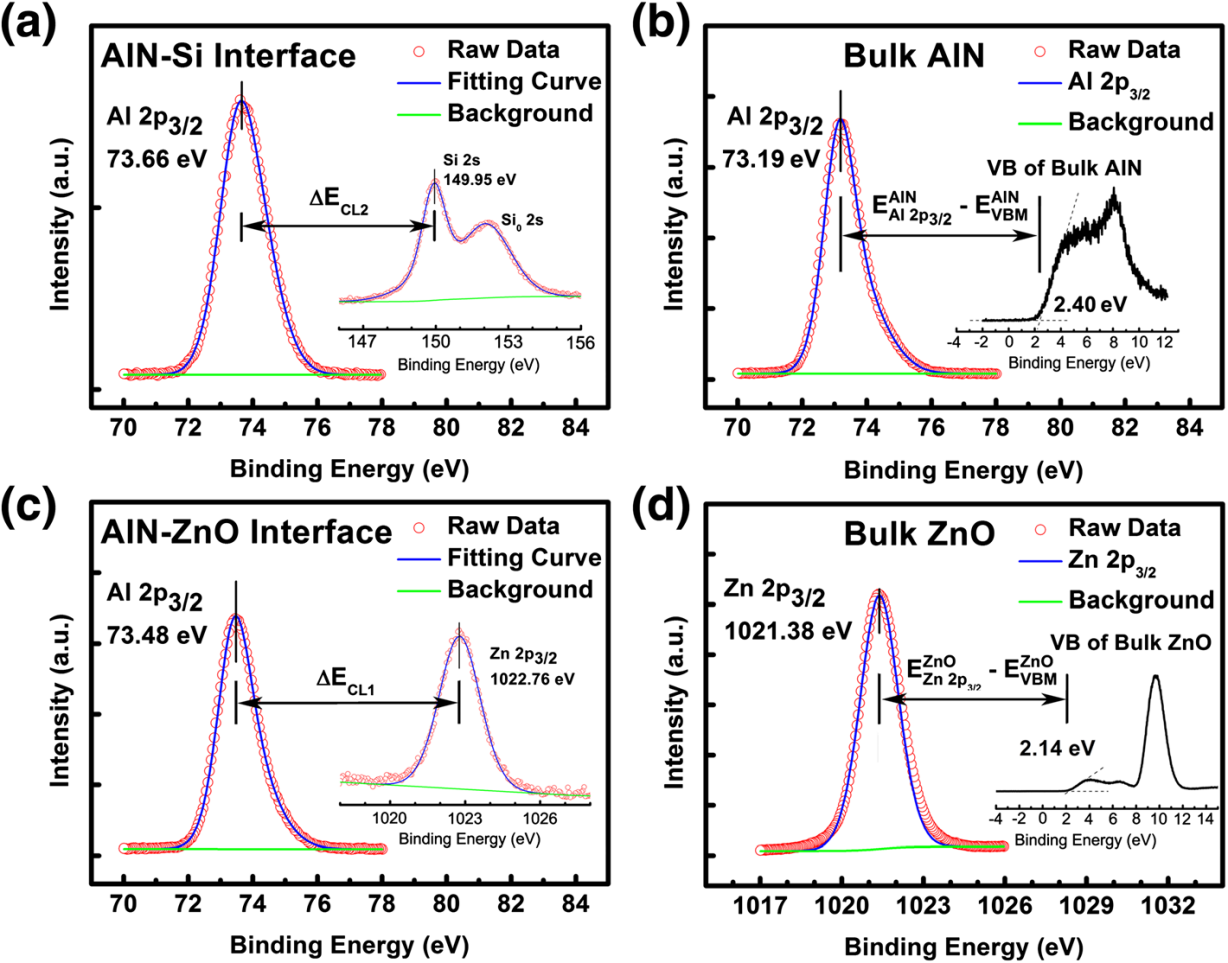
**XPS narrow scans of ZnO/AlN/Si heterojunction. (a)** XPS narrow scans of Al 2p3/2 and Si 2 s at AlN/Si interface; **(b)** XPS narrow scans of Al 2p3/2 and VBM of bulk AlN; **(c)** XPS narrow scans of Al 2p3/2 and Zn 2p3/2 at ZnO/AlN interface; **(d)** XPS narrow scans of Zn 2p3/2 and VBM of bulk ZnO on an AlN buffer layer.

The results of XPS measurements are shown in Figure 5 for ZnO/Si heterojunction and Figure 6 for ZnO/AlN/Si heterojunction. Figure 5a shows the VBM and the CL spectra of Si 2 s recorded on Si substrate. The VBM of bulk Si was determined to be 0.15 eV by linear extrapolation, and the Si 2 s peak was located at 150.18 eV corresponding to the Si-Si bond [16]. The Zn 2p_3/2_ and Si 2 s spectra at the interface of ZnO/Si heterojunction were presented in Figure 5b, and the peaks were located at 150.07 and 1022.26 eV, respectively. In the thick ZnO films, it can be seen from Figure 5c that the Zn 2p_3/2_ CL peak was slightly shifted to 1021.54 eV, corresponding to the Zn-O bonding state [27]. At the same time, the VBM of bulk ZnO was determined to be 2.38 eV. Thus, the *ΔE*CL was calculated to be 872.19 eV. $\varDelta {E}_{\mathrm{V}}^{\mathrm{ZnO}/\mathrm{S}\mathrm{i}}$ was then calculated to be 3.06 eV using Equation 1.

Figure 6a shows the Al 2p_3/2_ and Si 2 s spectra at the interface between AlN and Si. The Al 2p_3/2_ peak was located at 73.66 eV, corresponding to the Al-N bond [28]. Besides, the Si-Si bond at 149.95 eV, a significant peak appeared at 152.05 eV that can be attributed to the bonding configuration of Si-N [29]. This Si-N bond may originate from the nitridation process for the initial Si substrate processing performed at 850°C, forming SiN_x_ with an ultrathin thickness to be hardly measured. The VB and the CL spectra of Al 2p_3/2_ for the bulk AlN are displayed in Figure 6b. The VBM position and the Al 2p_3/2_ CL peak were determined to be 2.40 and 73.19 eV, respectively. In the meanwhile, the VBM and the Zn 2p_3/2_ CL were deduced to be 2.14 and 1021.38 eV for the bulk ZnO film with AlN buffer layer from the results shown in Figure 6d. Figure 6c shows the Al 2p_3/2_ and Zn 2p_3/2_ spectra at the interface of AlN/ZnO heterostructure. Compared to those of the bulk AlN and ZnO samples, the Al 2p_3/2_ and Zn 2p_3/2_ peaks shifted to 73.48 and 1022.76 eV, respectively. With all the values obtained above, $\varDelta {E}_{\mathrm{V}}^{\mathrm{AlN}/\mathrm{Z}\mathrm{n}\mathrm{O}}$ and $\varDelta {E}_{\mathrm{V}}^{\mathrm{AlN}/\mathrm{S}\mathrm{i}}$ were calculated to be −0.83 and 2.95 eV using Equations 2 and 3. Finally, the conduction band offset (*ΔE*C) at each interface can be estimated from the following formula:

4 $$ \varDelta {E}_{\mathrm{C}}^{\mathrm{ZnO}/\mathrm{S}\mathrm{i}}={E}_{{}_{\mathrm{g}}}^{\mathrm{ZnO}}-{E}_{\mathrm{g}}^{\mathrm{Si}}-\varDelta {E}_{\mathrm{V}}^{\mathrm{ZnO}/\mathrm{S}\mathrm{i}} $$

5 $$ \varDelta {E}_{\mathrm{C}}^{\mathrm{AlN}/\mathrm{S}\mathrm{i}}={E}_{{}_{\mathrm{g}}}^{\mathrm{AlN}}-{E}_{\mathrm{g}}^{\mathrm{Si}}-\varDelta {E}_{\mathrm{V}}^{\mathrm{AlN}/\mathrm{S}\mathrm{i}} $$

6 $$ \varDelta {E}_{\mathrm{C}}^{\mathrm{AlN}/\mathrm{Z}\mathrm{n}\mathrm{O}}={E}_{{}_{\mathrm{g}}}^{\mathrm{AlN}}-{E}_{\mathrm{g}}^{\mathrm{ZnO}}-\varDelta {E}_{\mathrm{V}}^{\mathrm{AlN}/\mathrm{S}\mathrm{i}} $$

The band gap of bulk ZnO, Si, and AlN at room temperature was known to be 3.37 [30], 1.12 [31], and 6.2 eV [19], respectively. With the VBO values that have already been calculated, the conduction band offsets (CBOs) were found as follows:

$$ \varDelta {E}_{\mathrm{C}}^{\mathrm{ZnO}/\mathrm{S}\mathrm{i}} = 0.81\ \mathrm{eV} $$, $$ \varDelta {E}_{\mathrm{C}}^{\mathrm{AlN}/\mathrm{S}\mathrm{i}} = 2.13\ \mathrm{eV} $$, and $$ \varDelta {E}_{\mathrm{C}}^{\mathrm{AlN}/\mathrm{Z}\mathrm{n}\mathrm{O}} = 3.66\ \mathrm{eV} $$.

**Figure 7:**
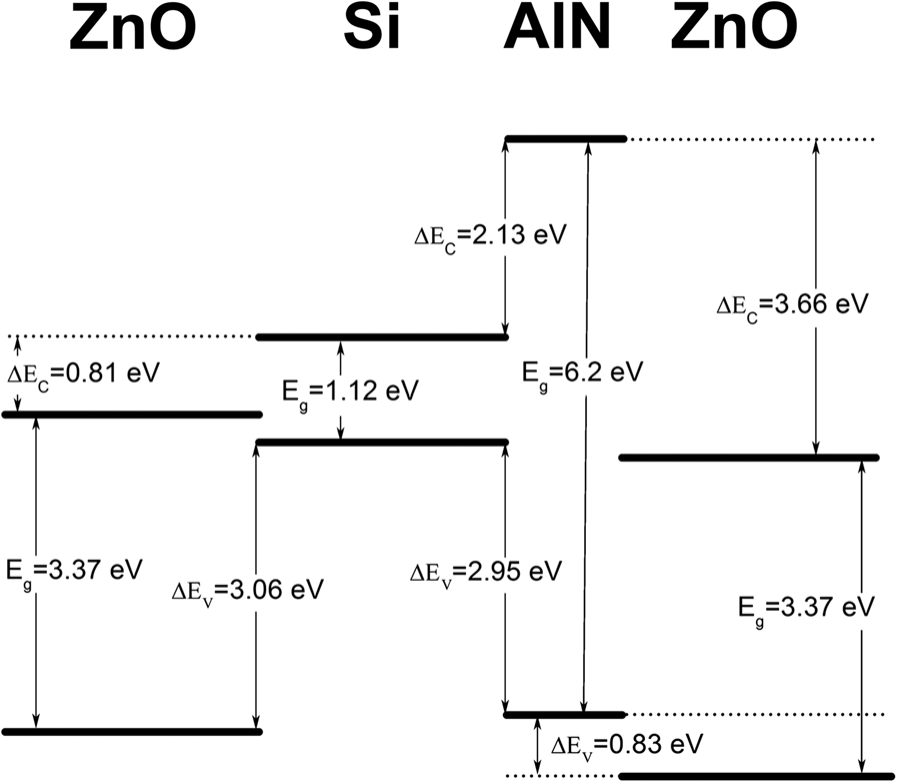
**Band alignment diagram of ZnO/Si and ZnO/AlN/Si bi-interface systems.**

The band alignment of two bi-interface systems is illustrated in Figure 7 which includes all energy levels.

As shown in Figure 7, the introduction of an AlN buffer layer lead to changes of VBOs and CBOs between ZnO and Si substrate. The presence of the AlN buffer layer can modify the band alignment between ZnO and Si to an extent as large as 0.72 eV. The VBO between AlN and Si substrate was smaller than that between ZnO and Si substrate, which makes it easier to inject holes from Si to ZnO by means of AlN. The AlN buffer layer acted as a ladder. More importantly, those tunneled holes need only overcome a 0.83 eV barrier to enter into ZnO. On the other side, the CBO (as large as 3.66 eV) between AlN and ZnO can block the flow of electrons from ZnO to p-Si. Thus, electron–hole recombination may mainly take place in the ZnO layer. A weak luminescence could be observed from the n-ZnO/AlN/p-Si heterojunction at room temperature when a positive voltage was applied to the p-Si substrate. However, more work need to be carried out to improve the luminous efficiency in the future.

## Conclusions

In summary, we had prepared heterojunctions of ZnO films on Si (111) substrates by ALD. The 60-nm-thick AlN buffer layer was introduced to improve the crystalline quality and change the growth direction of ZnO films from [100] to [002]. With the AlN buffer layers, the BET peak was enhanced while the DLE was still kept at a low level. The band alignment ZnO/Si heterojunction was modified to be 0.72 eV after introducing the AlN buffer layer. The large CBO between AlN/Si heterojunction blocks the flow of electrons from ZnO to p-Si, while the decreased VBO makes it easier to inject holes from p-Si to ZnO
